# Orai, STIM, and PMCA contribute to reduced calcium signal generation in CD8^+^ T cells of elderly mice

**DOI:** 10.18632/aging.102809

**Published:** 2020-02-12

**Authors:** Adrian Angenendt, Romy Steiner, Arne Knörck, Gertrud Schwär, Maik Konrad, Elmar Krause, Annette Lis

**Affiliations:** 1 Biophysics, Center for Integrative Physiology and Molecular Medicine, School of Medicine, Saarland University, Homburg 66421, Germany; 2 Molecular Biophysics, Center for Integrative Physiology and Molecular Medicine, School of Medicine, Saarland University, Homburg 66421, Germany; 3 Cellular Neurophysiology, Center for Integrative Physiology and Molecular Medicine, School of Medicine, Saarland University, Homburg 66421, Germany; 4 Present address: Section of Transplantation Immunology, Department of Surgery, Medical University of Vienna, Vienna 1090, Austria

**Keywords:** CD8 T cells, STIM, PMCA, calcium, Orai

## Abstract

Ca^2+^ is a crucial second messenger for proper T cell function. Considering the relevance of Ca^2+^ signals for T cell functionality it is surprising that no mechanistic insights into T cell Ca^2+^ signals from elderly individuals are reported. The main Ca^2+^ entry mechanism in T cells are STIM-activated Orai channels. Their role during lymphocyte aging is completely unknown. Here, we report not only reduced Ca^2+^ signals in untouched and stimulated, but also in central and effector memory CD8^+^ T cells from elderly (18-24 months) compared to adult (3-6 months) mice. Two mechanisms contribute to the overall reduction in Ca^2+^ signals of CD8^+^ T cells of elderly mice: 1) Reduced Ca^2+^ currents through Orai channels due to decreased expressions of STIMs and Orais. 2) A faster extrusion of Ca^2+^ owing to an increased expression of PMCA4. The reduced Ca^2+^ signals correlated with a resistance of the cytotoxic efficiency of CD8^+^ T cells to varying free [Ca^2+^]_ext_ with age. In summary, reduced STIM/Orai expression and increased Ca^2+^ clearing rates following enhanced PMCA4 expression contribute to reduced Ca^2+^ signals in CD8^+^ T cells of elderly mice. These changes are apparently relevant to immune function as they reduce the Ca^2+^ dependency of CTL cytotoxicity.

## INTRODUCTION

Aging of the immune system is a cumulative phenomenon and contributes to morbidity and mortality in man due to the greater incidence of infection, as well as autoimmune phenomena and cancer in elderly individuals [1–3]. Multiple changes in T cell populations are considered to be critical contributors to age-associated immune dysfunction [4]. A rather unexplored field of this dysfunction in aging is the Ca^2+^ homeostasis, which especially in T cells, is tightly regulated by an orchestra of channels, pumps, transporters, and receptors [5, 6]. The increase in cytoplasmic free [Ca^2+^] following T cell receptor activation is an essential element of various activation pathways [7] and necessary for the induction of gene expression in CD8^+^ T cells [8–11]. In addition, Ca^2+^ orchestrates the cytotoxicity of CD8^+^ T cells as the main function of this cell type [12]. For murine and human immune cells, store operated Ca^2+^ entry (SOCE) is the main pathway for Ca^2+^ to enter T cells [6, 13, 14]. Highly Ca^2+^-selective Orai channels and STIM proteins, as Ca^2+^ sensors, are the key players in SOCE and with their distinct characteristic properties [15, 16] they shape the Ca^2+^ pattern in T cells [17]. In contrast to the human SOCE components, four different Orai (Orai1, Orai2S, Orai2L, Orai3) proteins [18] and four STIM (STIM1, STIM1L, STIM2.1 and STIM2.2) proteins [19–21] have been identified in the mouse so far, but their role during aging in T cells is completely unknown.

While STIM/Orai channels are the likely candidates to mediate Ca^2+^ entry in aging T cells, other mechanisms could in principle contribute to store-operated Ca^2+^ signals: K^+^ channels (like K_v_1.3 and K_Ca_3.1) and TRPM4 may control the membrane potential and thereby the net Ca^2+^ entry through Orai channels [22, 23]. Furthermore, Ca^2+^ ATPases and mitochondria export Ca^2+^ from the cytosol and thereby shape Orai-dependent Ca^2+^ signals [24].

Following T cell activation in mice, several groups have reported a decline in Ca^2+^ signals with age [25, 26]. However, since the molecular mechanisms of these decreased Ca^2+^ signals in the context of aging are still elusive, we performed comprehensive assessments of the molecular repertoire governing Ca^2+^ signals in CD8^+^ T cells and investigated the influence of varying [Ca^2+^]_ext_ on the main function of these cells, the lysis of target cells.

## RESULTS

### Elderly mice show an increased proportion of CD8^+^ T cells and a shift in subpopulations

First, we examined the health status of the mice used for experiments. For this, body weight and spleen weight were measured (Table 1). Splenomegaly is a frequently observed sign of non-specific necropsy [27], therefore we excluded those mice from our cohort. A shift in the distribution of T cell subtypes to less naïve and more memory cells is considered as a marker of immunosenescence [26, 28, 29]. To assess the CD8^+^ T cell subtype distribution of the cells we stained them with surface markers CD44 and CD62L to differentiate between naïve (N: CD62L^high^CD44^low^); effector memory (EM: CD62L^low^CD44^high^), and central memory CD8^+^ T cells (CM: CD62L^high^CD44^high^) (Supplementary Figure 1A, 1D). The untouched cells of adult mice display a pronounced population of CD62L^high^CD44^low^ naϊve cells, a lesser relative amount of CD62L^high^CD44^high^ central memory cells and only a small proportion of CD62L^low^CD44^high^ effector memory cells (Supplementary Figure 1A, 1C). In contrast, the untouched cells of elderly mice only show a small population of naϊve cells, a prominent population of effector and an even bigger population of central memory cells (Supplementary Figure 1D, 1F). The stimulation with CD3/CD28 beads led to a significant loss of the naïve population of CD8^+^ T cells and a significant increase in the relative amount of effector memory cells for both the adult and the elderly age group (examples in Supplementary Figure 1B, 1E, statistics in Supplementary Figure 1C, 1F). Furthermore, the population of central memory cells showed a significant increase for the CD8^+^ T cells of adult and a significant decrease for the CD8^+^ T cells of elderly mice after stimulation. In summary, the CD8^+^ T cells of adult mice mainly consist of naϊve cells and shifts primarily to central and secondarily to effector memory cells, whereas the CD8^+^ T cell pool of elderly mice mostly consists of memory T cells and shifts to more effector memory cells with T cell activation.

**Table 1:** Spleen to body weight ratio of adult (n = 91) and elderly (n = 71) mice ± SEM.

	**body weight (g)**	**spleen weight (mg)**	**spleen to body weight ratio (%)**
**adult**	24.65 (± 0.24)	93.70 (± 1.59)	0.380 (± 0.005)
**elderly**	33.10 (± 0.58)	139.41 (± 3.53)	0.425 (± 0.011)

### Untouched CD8^+^ T cells from elderly mice show reduced SOCE

**Figure 1:**
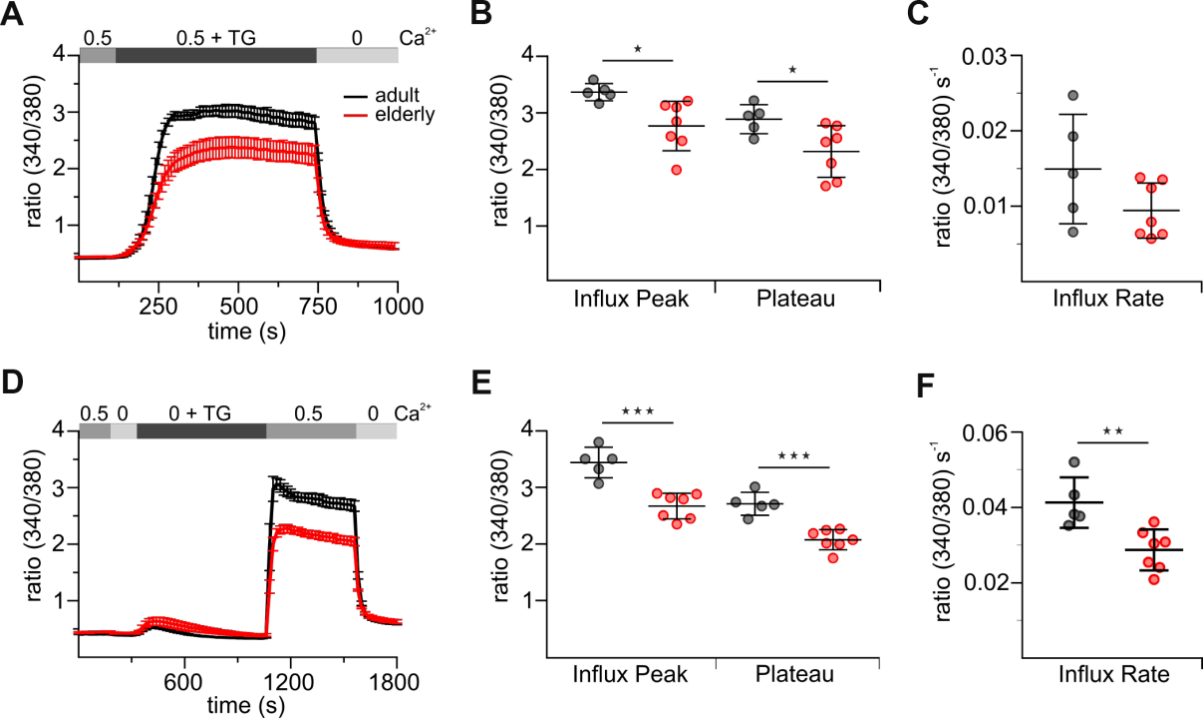
**Untouched CD8^+^ T cells from elderly mice show reduced thapsigargin (TG)-induced Ca^2+^ signals.** (**A**) Fura2-AM based Ca^2+^ Imaging with 1 μM TG as stimulus applied in the presence of 0.5 mM [Ca^2+^]_ext_ of CD8^+^ T cells (combined Ca^2+^ protocol) from adult (black, n = 5) and elderly (red, n = 7) mice. The scatter dot plot in (**B**) displays the corresponding statistics of Ca^2+^ influx peak and Ca^2+^ plateau and in (**C**) the corresponding influx rates. (**D**) Ca^2+^ Imaging with 1 μM TG applied in the absence of [Ca^2+^]_ext_ before re-addition of 0.5 mM Ca^2+^ (re-addition protocol) of CD8^+^ T cells from adult (black, n = 5) and elderly (red, n = 7) mice. The scatter dot plot in (**E**) displays the corresponding statistics of Ca^2+^ influx peak and Ca^2+^ plateau and (**F**) the corresponding influx rates. Ca^2+^ signalling curves are presented as mean ± SEM. Scatter dot plots are presented as mean ± SD. * p < 0.05, ** p < 0.01, *** p < 0.001, **** p < 0.0001.

Ca^2+^ influx is an essential step in T cell activation and regulation of diverse cellular functions and the main pathway of Ca^2+^ in immune cells is through STIM-gated Orai channels [5]. We first investigated store-operated Ca^2+^ responses of elderly and adult untouched CD8^+^ T cells activated by thapsigargin (TG), an irreversible inhibitor of the sarco/endoplasmic reticulum Ca^2+^-ATPase (SERCA) pumps. We measured Ca^2+^ mobilization with two different approaches. The first approach combined the measurement of Ca^2+^ release and entry (combined protocol, Figure 1A) in the presence of 0.5 mM [Ca^2+^]_ext_, whereas the second one separates them (Figure 1D) by carrying out the classical Ca^2+^ re-addition protocol. In the latter protocol, cells are initially treated with TG in Ca^2+^-free Ringer’s solution to deplete the endoplasmic reticulum (ER) Ca^2+^ stores, and Ca^2+^ influx is assured by subsequent addition of Ca^2+^. CD8^+^ T cells from elderly and adult mice differ in their ability to increase the internal calcium concentration ([Ca^2+^]_int_) after TG-triggered stimulation in both approaches (Figure 1A, 1D). Resting [Ca^2+^]_int_ in CD8^+^ T cells from both mice was not significantly changed in combined and re-addition protocol (Figure 1A, 1D, Supplementary Tables 1, 2). Treatment of cells with TG in the absence of [Ca^2+^]_ext_ resulted in a transient rise in [Ca^2+^]_int_ revealing no significant change in the size of TG-releasable peak (Figure 1D, Supplementary Table 2). Upon re-addition of [Ca^2+^]_ext_ but also with the combined Ca^2+^ protocol, [Ca^2+^]_int_ mobilization was reduced in elderly CD8^+^ T cells by about 25 % (Figure 1A, 1B, 1D, 1E, Supplementary Tables 1, 2). The rate of Ca^2+^ entering CD8^+^ T cells is an indirect read-out for Orai channel function. For the re-addition protocol, the Ca^2+^ entry rate was significantly slower in cells from elderly compared to adult mice (Figure 1F); a similar tendency was observed in the combined protocol (Figure 1C). Similar results were obtained in case [Ca^2+^]_ext_ was increased to 2 mM (Supplementary Figure 2, Supplementary Tables 3, 4).

### Reduced I_CRAC_ in untouched CD8^+^ T cells from elderly mice

**Figure 2:**
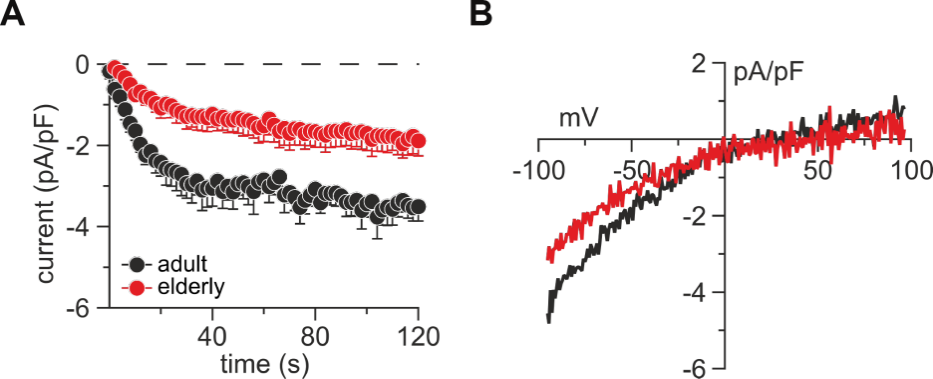
**Untouched CD8^+^ T cells from elderly mice show reduced lP_3_-induced CRAC currents.** (**A**) Average IP_3_-induced CRAC current amplitudes at –80 mV normalized to cell size from CD8^+^ T cells of adult (black, n = 8) and elderly (red, n = 7) mice. (**B**) Average current-voltage relationship of CRAC currents from cells presented in (**A**) after CRAC had fully developed. Data obtained are presented as mean ± SEM.

Since mainly Ca^2+^ release-activated Ca^2+^ currents (I_CRAC_) through Orai channels are responsible for Ca^2+^ influx in T cells, we assessed I_CRAC_ in untouched CD8^+^ T cells from adult and elderly mice to determine age-related changes (Figure 2). We performed whole-cell patch-clamp experiments by perfusing cells with a high concentration of the Ca^2+^ chelator BAPTA, and IP_3_ to deplete the Ca^2+^ stores. Both adult and elderly CD8^+^ T cells immediately developed a small, inwardly rectifying current (I_CRAC_) with a reversal potential above +40 mV. Elderly CD8^+^ T cells showed reduced currents by 46 % (-1.89 ± 0.37 compared with -3.51 ± 0.36 (pA/pF) at 120 sec, p < 0.0001) (Figure 2A) with the typical inwardly rectifying current-voltage relationship (IV, Figure 2B).

### Reduced store-operated Ca^2+^ entry in in vitro stimulated CD8^+^ T cells from elderly mice

**Figure 3:**
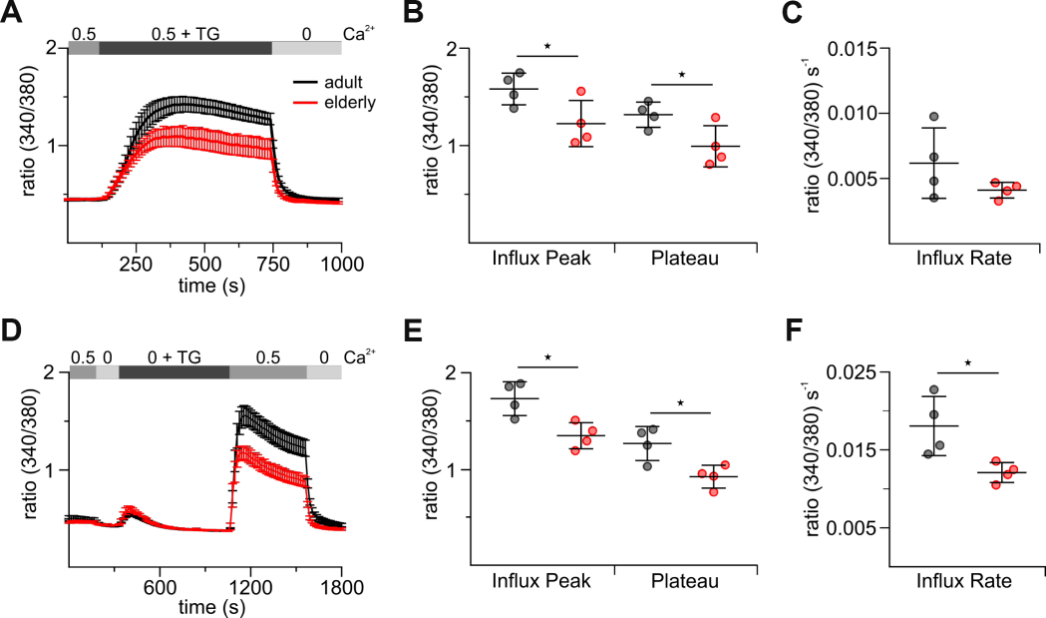
**Stimulated CD8^+^ T cells from elderly mice show reduced thapsigargin (TG)-induced Ca^2+^ signals.** (**A**) Fura2-AM based Ca^2+^ Imaging with 1 μM TG as stimulus applied in the presence of 0.5 mM [Ca^2+^]_ext_ of CD8^+^ T cells (combined Ca^2+^ protocol) from adult (black, n = 4) and elderly (red, n = 4) mice. The scatter dot plot in (**B**) displays the corresponding statistics of Ca^2+^ influx peak and Ca^2+^ plateau and in (**C**) the corresponding influx rates. (**D**) Ca^2+^ Imaging with 1 μM TG applied in the absence of [Ca^2+^]_ext_ before re-addition of 0.5 mM Ca^2+^ (re-addition protocol) of CD8^+^ T cells from adult (black, n = 4) and elderly (red, n = 4) mice. The scatter dot plot in (**E**) displays the corresponding statistics of Ca^2+^ influx peak and Ca^2+^ plateau and (**F**) the corresponding influx rates. Ca^2+^ data are presented as mean ± SEM. Scatter dot plots are presented as mean ± SD. * p < 0.05, ** p < 0.01, *** p < 0.001, **** p < 0.0001.

Since the negatively isolated and untouched cells do not have a long life span (24 hours) and, just like virus-infected or cancer cells, stimulation leads to activated CD8^+^ T cells, we decided to validate Ca^2+^ signals in CD8^+^ T cells after *in* *vitro* stimulation. We therefore stimulated the CD8^+^ T cells with anti-CD3/CD28 stimulation beads and examined SOCE on day 3 after stimulation. The overall Ca^2+^ signals analyzed in combined and re-addition protocols were reduced in stimulated CD8^+^ T cells between 60 to 64 % compared to untouched cells (Figures 1A, 1D, 3A, 3D, Supplementary Table 1, 2). This suggested that the molecular composition of the CRAC channel and STIM sensors may change during T cell stimulation. Still, TG-induced SOCE, measured as a peak of the Ca^2+^ response was significantly reduced in stimulated elderly CD8^+^ T cells compared to adult as control (Figure 3B, 3E). Besides the peak, also the Ca^2+^ plateau, as an important determinant of Ca^2+^ dependent cellular responses, was reduced in elderly CD8^+^ T cells (Figure 3B, 3E). For the re-addition protocol, the Ca^2+^ entry rate was significantly slower in cells from elderly compared to adult mice (Figure 3F); a similar tendency was observed in the combined protocol (Figure 3C). In contrast to untouched CD8^+^ T cells, the application of 2 mM [Ca^2+^]_ext_ was able to rescue the impaired Ca^2+^ signal in the elderly CD8^+^ T cells at least to some extend (Supplementary Figure 3). Measurements of I_CRAC_ in CD3/CD28 bead-stimulated CD8^+^ T cells were not successful due to their already overall small whole-cell currents that were presumably even more reduced in the T cells from elderly mice.

### CD8^+^ T cells from elderly mice show reduced Ca^2+^ signals after T cell receptor stimulation and are less affected in their cytotoxic function by varying free external Ca^2+^ concentrations

**Figure 4:**
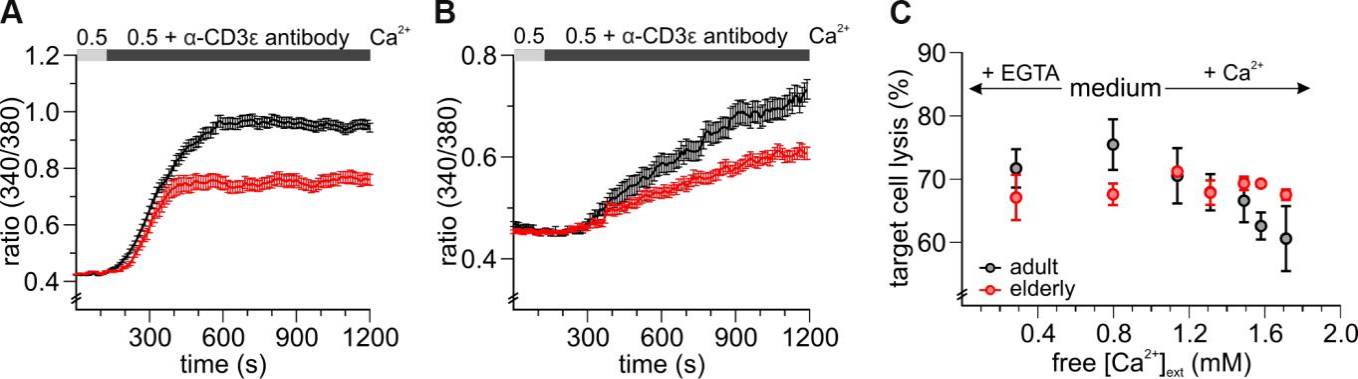
**CD8^+^ T cells from elderly mice show reduced Ca^2+^ signals after T cell receptor stimulation and are less affected in their cytotoxic function by varying free external Ca^2+^ concentrations.** Fura2-AM based Ca^2+^ Imaging with 2 μg/ml anti-CD3 antibody as stimulus applied in the presence of 0.5 mM [Ca^2+^]_ext_ of (**A**) untouched (black: adult, n = 664 cells; red: elderly, n = 327 cells) and (**B**) stimulated (black: adult, n = 155 cells; red: elderly, n = 116 cells) CD8^+^ T cells from adult and elderly mice. (**C**) The cytotoxic function of CD8^+^ T cells from elderly mice is less affected by varying free [Ca^2+^]_ext_. Changes in end-point lysis with the addition of Ca^2+^ or the Ca^2+^ chelating agent EGTA to the medium of a cytotoxicity assay for CD8^+^ T cells of adult (grey, n = 3 - 5) and elderly (n = 2 - 3) mice. Data obtained are presented as mean ± SEM.

To test for a functional relevance of reduced [Ca^2+^]_int_ we investigated SOCE in response to a more physiological stimulus. Antibody binding to the CD3/T-cell receptor complex activates T cells and evokes Ca^2+^ signals [30]. To explore the differences in TCR-induced [Ca^2+^]_int_ mobilization between adult and elderly CD8^+^ T cells we activated the TCR by application of a soluble anti-CD3ε antibody. Figure 4 shows that TCR activation leads to increased Ca^2+^ influx in untouched (Figure 4A) and stimulated (Figure 4B) CD8^+^ T cells but could not reach the levels seen in TG-experiments (Figure 1A, 3A). Mean [Ca^2+^]_int_ mobilization of the untouched cells was faster and reached overall a higher plateau compared to the stimulated counterparts. As in TG-induced SOCE, CD8^+^ T cells isolated from elderly mice show less efficient TCR-induced [Ca^2+^]_int_ mobilization compared to adult mice.

The characteristic function of CD8^+^ T cells is to eliminate virus-infected and degenerated targets cells [31]. In our previous paper, we reported how changes in [Ca^2+^]_ext_ influence human CD8^+^ T cell cytotoxicity [12]. Therefore, we investigated, whether different concentrations of [Ca^2+^]_ext_ have an impact on the killing efficiency of CD8^+^ T cells in the context of aging. Either EGTA or CaCl_2_ were added to AIM V, a medium optimized for the cultivation of lymphocytes, to decrease or increase free [Ca^2+^]_ext_. Quantification of cancer cell killing revealed that free [Ca^2+^]_ext_ higher or lower than the value of 798 μm of AIM V reduced the cytotoxicity of CD8^+^ T cells of adult mice (Figure 4C) similarly as for human CD8^+^ T cells [12]. In contrast, CD8^+^ T cells of elderly mice where largely unaffected by fluctuations in free [Ca^2+^]_ext_ (Figure 4C). Averaging all experiments showed that elderly CD8^+^ T cells showed similar cytotoxic efficiency against target cells at a relatively wide range of free [Ca^2+^]_ext_ between 74 up to 1715 μM compared to their adult counterpart. Thus, Ca^2+^ dependent regulation of cytotoxicity is reduced in CD8^+^ cells from elderly mice.

### STIM and Orai molecules are differently expressed in untouched and stimulated CD8^+^ T cells from elderly mice

**Figure 5:**
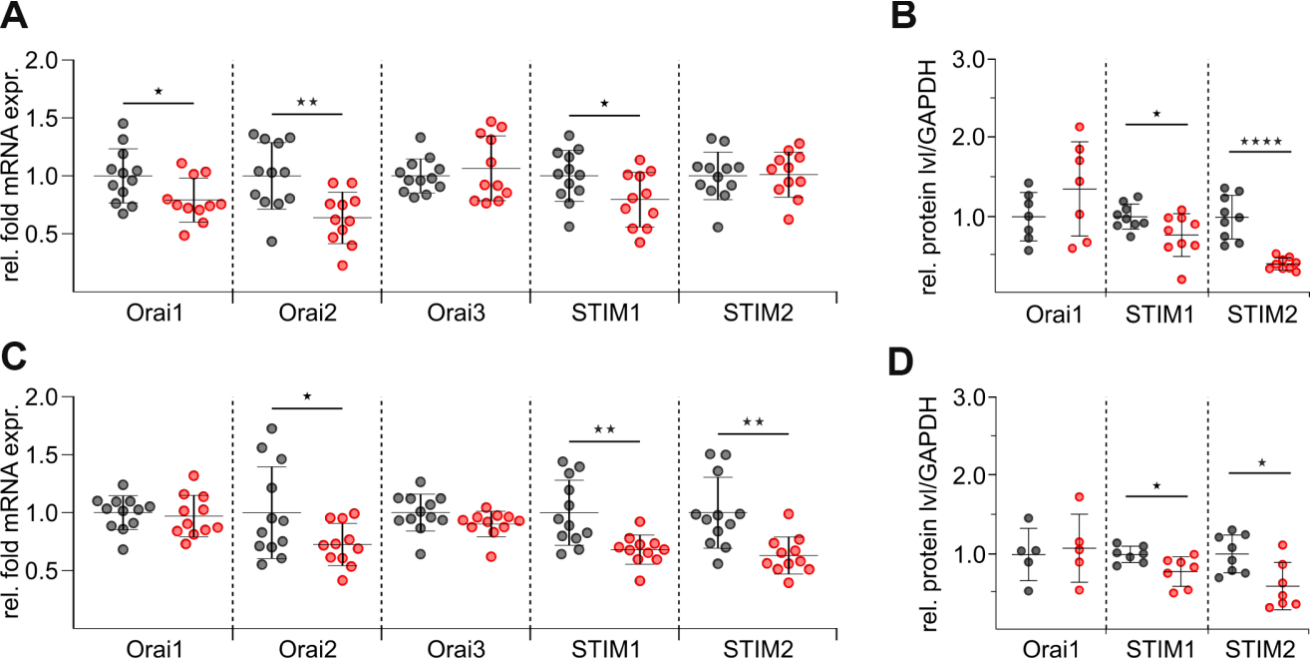
**mRNA and protein levels of distinct STIM and Orai isoforms are reduced in CD8^+^ T cells of elderly mice.** (**A**) Normalised relative mRNA expressions of Orai1, 2 and 3 and STIM1 and 2 of untouched CD8^+^ T cells from adult (grey, n = 12) and elderly (red, n = 11) mice. (**C**) Normalised relative mRNA expressions of SOCE components of stimulated CD8^+^ T cells from adult (grey, n = 12) and elderly (red, n = 11) mice. Protein quantification after normalization to GAPDH of SOCE components from (**B**) untouched (n = 7 - 9) and (**D**) stimulated (n = 5 - 7) CD8^+^ T cells lysates isolated from adult (black) and elderly (red) mice. Data obtained are presented as mean ± SD. * p < 0.05, ** p < 0.01, *** p < 0.001, **** p < 0.0001.

The Ca^2+^ influx profile in lymphocytes is strictly determined by the composition of Orai channels and STIM Ca^2+^ sensors [5, 32, 33]. The ratio of Orai to STIM determines the characteristics and properties of I_CRAC_ [34, 35]. Therefore, we performed a detailed analysis of the expression levels of Orais (Orai1, 2, and 3) and STIMs (STIM1 and 2) in untouched and stimulated CD8^+^ T cells from adult and elderly mice by quantitative real-time PCR and western blot analysis. As expected, mRNA of both STIMs and all three Orais are abundantly expressed in untouched (Figure 5A) and stimulated (Figure 5C) CD8^+^ T cells from both age groups. To facilitate comparison, expression levels of Orai and STIM genes from elderly mice were normalized to reference genes and shown as relative fold change to the adult group. The untouched CD8^+^ T cells from elderly mice showed a significant reduction in mRNA transcript levels for Orai2 and STIM1 (Figure 5A), which could also be confirmed at protein levels after densitometry analysis (Figure 5B, Supplementary Figure 4C). Surprisingly, as shown in Figure 5A, mRNA levels of STIM2 were not changed in untouched CD8^+^ T cells of elderly mice but the statistical analysis of the western blots revealed a significant reduction on protein levels (Figure 5B). The mRNA reduction of Orai1 (Figure 5A) could not be confirmed at the protein level (Figure 5B). In addition, a significant downregulation for STIM1 and STIM2 was seen in stimulated CD8^+^ T cells from elderly mice at mRNA transcript and protein levels (Figure 5C, 5D). The level of Orai3 mRNA remained unchanged in untouched and stimulated cells (Figure 5A, 5C). Protein analysis of Orai2 or Orai3 was hampered by limited sensitivity of commercially available antibodies. Since the patch-clamp attempts with CD3/CD28 bead-stimulated CD8^+^ T cells from adult and elderly mice were unsuccessful, we were wondering if this is due to the reduced levels of Orai and STIM after stimulation. Indeed, the stimulated CD8^+^ T cells showed a significant downregulation of all STIM and Orai mRNA transcript levels compared to the untouched CD8^+^ T cells for both age groups (Supplementary Figure 4A, 4B). In conclusion, the overall expression levels of STIM and Orai correlate well with the reduction of Ca^2+^ entry in CD8^+^ cells from elderly compared to adult cells and also with the reduction of Ca^2+^ signals in stimulated compared to untouched cells.

### Enhanced Ca^2+^ clearance rate in CD8^+^ T cells from elderly mice

**Figure 6:**
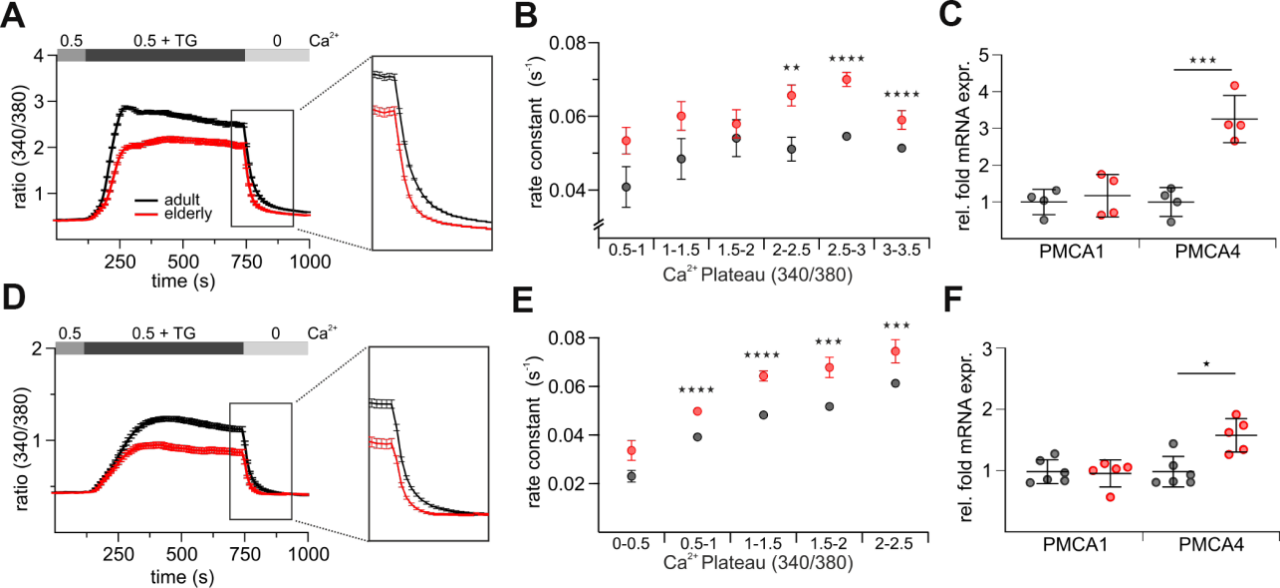
**CD8^+^ T cells from elderly mice show a faster efflux of Ca^2+^.** Exemplary combined protocol measurement of untouched (**A**) and stimulated (**D**) CD8^+^ T cells with a highlight on the Ca^2+^ plateau and efflux. Rate constants of untouched (**B**) and stimulated (**E**) CD8^+^ T cells from adult and elderly mice plotted against their respective Ca^2+^ plateaus. (**C**) Relative mRNA expressions of PMCA1 and 4 of untouched CD8^+^ T cells from adult (grey, n = 7) and elderly (red, n = 7) mice. (**F**) Relative mRNA expressions of PMCA1 and 4 of stimulated CD8^+^ T cells from adult (grey, n = 7) and elderly (red, n = 8) mice. Data obtained are presented as mean ± SD. * p < 0.05, ** p < 0.01, *** p < 0.001, **** p < 0.0001.

In addition to the influx mechanisms, [Ca^2+^]_int_ of course depends on Ca^2+^ efflux that could contribute to the difference in Ca^2+^ signals of CD8^+^ T cells from adult and elderly mice. We therefore compared the clearance rates of both untouched (Figure 6A) and stimulated (Figure 6D) cells from elderly and adult mice. Because the rate of Ca^2+^ extrusion in T cells depends on [Ca^2+^]_int_ [36, 37], steady-state [Ca^2+^]_int_ was measured just before Ca^2+^ removal. The clearance rates were directly calculated as exponential decays after removal of [Ca^2+^]_ext_. To isolate the effect of [Ca^2+^]_int_ we performed iso-cell analysis [37, 38] to compare the rate constant at approximately the same levels of [Ca^2+^]_int_. The rate constants from untouched (Figure 6B) and stimulated (Figure 6E) CD8^+^ T cells isolated from adult and elderly mice were plotted against the respective Ca^2+^ plateaus. Both the untouched and the stimulated CD8^+^ T cells from the elderly mice show significantly faster Ca^2+^ extrusion rates compared to the iso-cells from the adult mice (Figures 6B, 6E, Supplementary Table 5). PMCA1 and PMCA4 are the two, out of four, known PMCA isoforms that are ubiquitously distributed in all tissues and they are also the major Ca^2+^ extrusion pathway in T cells [37]. PMCA4b is highly expressed in T cells and important to shape [Ca^2+^]_int_ in T cells [37]. It may therefore contribute to the higher Ca^2+^ clearance rate seen in elderly CD8^+^ T cells. We tested the mRNA expression levels of PMCA1 and 4 in untouched and stimulated CD8^+^ T cells from adult and elderly mice (Figure 6C, 6F). In agreement with the Ca^2+^ clearance rate analysis, PMCA4b expression was increased in CD8^+^ cells from elderly compared to adult mice; the values for the PMCA1 isoform remained constant (Figure 6C, 6F). In conclusion, PMCA4b upregulation in CD8^+^ T cells from elderly mice might contribute to the observed phenotype of reduced [Ca^2+^]_int_.

### Altered SOCE in central and effector memory CD8^+^ T cells from elderly mice

**Figure 7:**
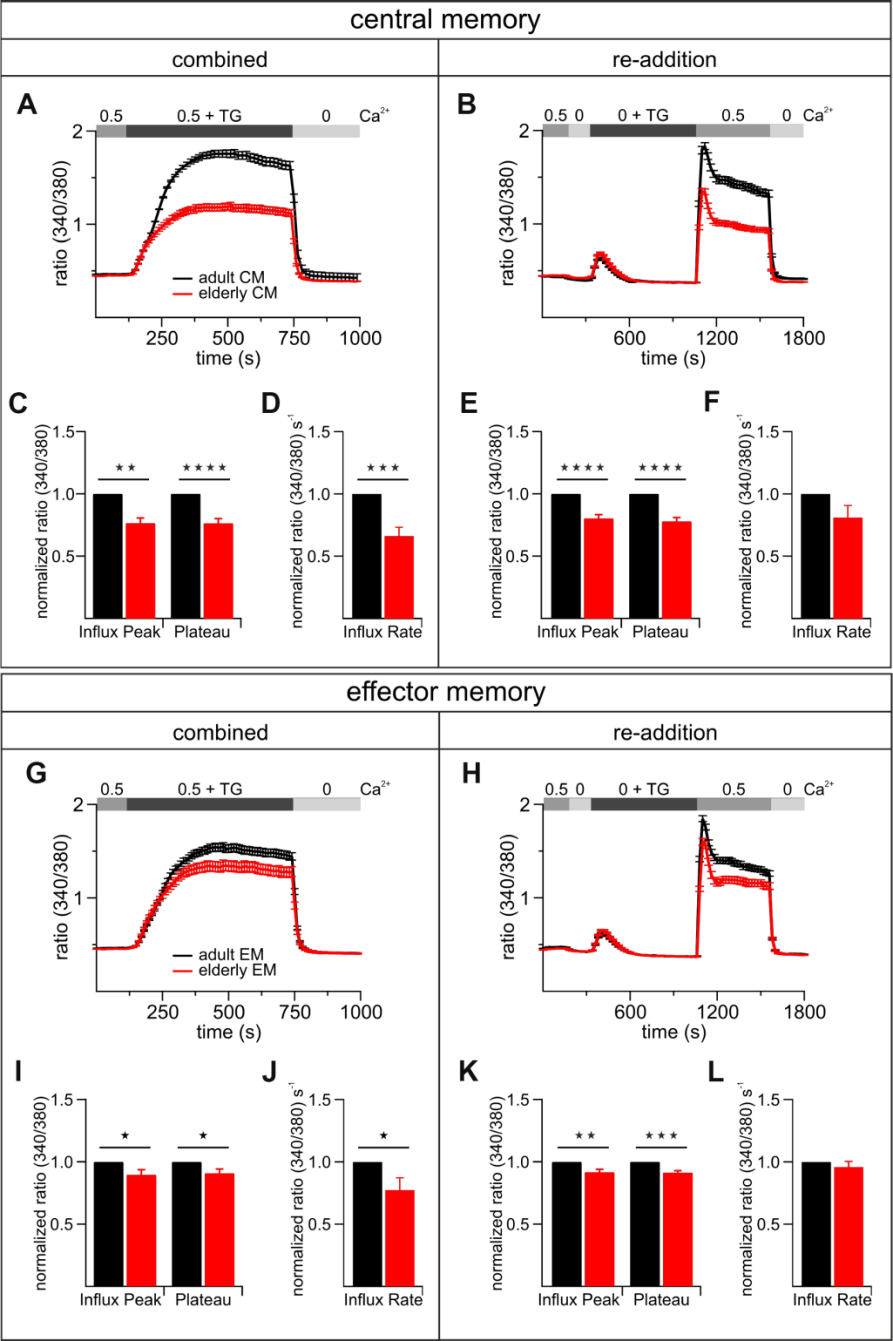
**The most abundant subtypes of activated CD8^+^ T cells exhibit age-related reductions of TG-induced Ca^2+^ signals.** Combined (**A**) and re-addition (**B**) protocol of CD8^+^ central memory T cells (CM) from adult (black, n = 6) and elderly (red, n = 6) mice. The bar graphs in (**C**) and (**E**) display the corresponding statistics of Ca^2+^ influx peak and Ca^2+^ plateau and in (**D**) and (**F**) the corresponding influx rates of combined and re-addition protocol, respectively. Combined (**G**) and re-addition (**H**) protocol of CD8^+^ effector memory T cells (EM) from adult (black, n = 6) and elderly (red, n = 6) mice. The scatter dot plots in (**I**) and (**K**) display the corresponding statistics of Ca^2+^ influx peak and Ca^2+^ plateau and in (**J**) and (**L**) the corresponding influx rates of combined and re-addition protocol, respectively. Ca^2+^ signalling curves show one exemplary out of six measurements with equal tendencies of central and effector memory cells of adult and elderly mice as mean ± SEM. Bar graphs show values of CD8^+^ T cells from elderly mice normalized to the values of CD8^+^ T cells from adult mice as mean ± SEM. * p < 0.05, ** p < 0.01, *** p < 0.001, **** p < 0.0001.

While changes in the (relative) frequency of distinct T cell subsets have been described, nothing is known about Ca^2+^ signal differences between subsets and if these explain the differences in the total population of CD8^+^ T cells from elderly and adult mice. In order to characterize the [Ca^2+^]_int_ mobilization of the most abundant subtypes of stimulated CD8^+^ T cells, we performed FACS sorting to separate the subtypes of adult and elderly mice and measured their Ca^2+^ signals. The normalized data of the adult CD8^+^ T cells subpopulations is summarized in Figure 7. Overall the [Ca^2+^]_int_ reduction of CD8^+^ T cells from elderly compared to adult mice is more prominent in the central memory (CM) than the effector memory (EM) population (Figure 7). The CM from elderly mice showed a more drastic Ca^2+^ influx peak reduction (20 - 25 %) (Figure 7A–7C, 7E) than the EM (~ 10 %) (Figure 7G–7I, 7K). The same applies for the Ca^2+^ plateaus of CM (~ 25 %) (Figure 7A–7C, 7E) and EM (~ 10 %) (Figure 7G–7I, 7K). The influx rates of both CM and EM showed a significant reduction for the more physiological combined protocol (CM: ~ 35 %; EM: ~ 23 %) (Figure 7D, 7F, 7H, 7J). In conclusion, from the two predominant subtypes of stimulated CD8^+^ T cells, the CM from elderly mice revealed a more distinctly reduced overall Ca^2+^ response compared to those of adult animals (Figure 7A, 7B). For the EM this age-related reduction was less pronounced (Figure 7G, 7H). Considering the percentage distribution of the subpopulations in both age cohorts (Supplementary Figure 1), the CM/EM ratios reflect the Ca^2+^ signal differences seen in the total population of CD8^+^ T cells (Figure 3A, 3D).

## DISCUSSION

Proper Ca^2+^ homeostasis is essential for the immune system to act fast and specific to eliminate tumor cells at early stages. Altered Ca^2+^ fluctuations have already been associated with numerous age-related diseases, such as neurodegenerative [39–41], autoimmune and inflammatory disorders [42]. Ca^2+^ responses are regulated negatively and positively by several mechanisms involving channels, pumps and sensors [43]. During aging, a continuous and steady decrease of all immune function takes places [2, 44–46] and especially for T cells alterations in Ca^2+^ homeostasis have been reported [25, 26].

Although Ca^2+^ is an important second messenger the underlying cause or mechanism of Ca^2+^ defects at molecular level during aging are not well understood and remain elusive. In this study, we perform for the first time a detailed analysis of Ca^2+^ signals in CD8^+^ T cells from elderly mice and link the aging-related reduction in Ca^2+^ signals to reductions of the main key players in the Ca^2+^ signaling pathway, further leading to coherent, concurrent functional alterations in Ca^2+^ homeostasis. Specifically, we found: 1) reduction of the expression of STIM and Orai proteins leads to reduced Ca^2+^ entry; 2) upregulation of PMCA4 additionally contributes to faster Ca^2+^ extrusion 3) less efficient TCR-induced [Ca^2+^]_int_ mobilization and 4) increased insensitivity to Ca^2+^ fluctuations during cytotoxic activity. These findings likely account for the observed reduced Ca^2+^ signals in CD8^+^ T cells from elderly mice and contribute to the declined T cell responses.

Generally, one would rather expect an overall reduction in expression of the key proteins involved in the Ca^2+^ homeostasis during aging. However, this is not what we observed in CD8^+^ T cells: whereas Orai1 and STIM1/2 proteins are downregulated, PMCA4b is in contrast upregulated. Both expression changes, however, result in reduced [Ca^2+^]_int_. Thus, it can be speculated that there is a deliberate change or adjustment in CD8^+^ T cells to reduced Ca^2+^ signals during the aging process. The age-related reductions in CD8^+^ T cell Ca^2+^ signals observed in our elderly mice may not imperatively implicate flawed cellular pathways and functions. Hence, one of the fundamental questions is how CD8^+^ T cells may benefit from reduced Ca^2+^ entry in age?

For the lysis of target cells, CD8^+^ cytotoxic T Lymphocytes (CTL) require a sequence of programmed steps, including target cell binding (conjugate formation), delivery of the lethal hit, target cell lysis, and killer cell recycling [47–49]. Several of these steps in the CTL killing machinery require or are modulated by Ca^2+^ itself [13]. It is well conceivable that Ca^2+^ fluctuations may greatly influence target cell killing by CTL and NK cells, and CRAC channels are well-suited to modulate killing because their dissociation constant K_D_ for Ca^2+^ permeation is in the range of 0.84 and 1.17 mM [50, 51]. Recently, we analyzed the cytotoxicity of human CTL and NK cells against cancer cells [12]. CTLs showed Ca^2+^ dependent cytotoxicity with an optimum for cancer cell elimination at rather low free [Ca^2+^] concentrations. Downregulation of Orai1 in CTLs led to decreased Ca^2+^ signals and increased efficiency to eliminate cancer cells [12]. Alterations of the STIM:Orai stoichiometry might regulate the killing efficiency of CD8^+^ T cells by changing the cells Ca^2+^ signals to be closer to or further away from the Ca^2+^ optimum for target cell killing. In the context of aging, we were able to detect changes at the mRNA levels in untouched and stimulated CD8^+^ T cells but not all Orai and STIM are affected in the same way. While Orai3 has hardly changed, decreased mRNA levels for Orai1, STIM1 and STIM2 could also be confirmed at the protein levels (Figure 5, Supplementary Figure 4). Surprisingly, the data for STIM2 showed no tendency at mRNA levels for the untouched CD8^+^ T cells (Figure 5A) but a significant decrease at protein levels (Figure 5B). Interestingly, also for Orai1 we observed a slight but not significant increase on protein level (Figure 5B) while the mRNA levels were significantly decreased (Figure 5A). This discrepancy may indicate a change in the turnover rate [52], translational regulation [53] or posttranslational modification [54] of these proteins. The stoichiometry of the STIM:Orai ratio determines the current size and inactivation properties [34, 35, 55] and thus Ca^2+^ signals and cell-specific cellular responses. Therefore, small variations in external Ca^2+^ could significantly alter Ca^2+^ signals and Ca^2+^-dependent target cell killing. Surprisingly, CD8^+^ T cells of elderly mice were mostly unaffected by fluctuations in free [Ca^2+^]_ext_ during killing compared to the CD8^+^ T cells from adult mice (Figure 4C). This independence from external Ca^2+^ fluctuations could be an advantage for the per se impaired CD8^+^ T cells from elderly mice. This insusceptibility to the wide range in free [Ca^2+^]_ext_ may offer an excellent adaptation to constantly changing surroundings such as the tumor microenvironment [56].

Additionally, there is no doubt about the importance of Ca^2+^ for cell proliferation not only for immune cells but also in the context of cancer cells [57–60]. Increased Ca^2+^ levels lead to an increased proliferation rate and SOCE plays a central role in its regulation [61]. However, the role of Orai channels in proliferation seems to be more complex considering the different results for different mouse models used for the investigation on this matter [62, 63]. Deletion of Orai [62] as well as STIM1 in T cells did not alter the proliferation, while T cells lacking both STIM1 and STIM2 proliferate to a much lesser extent [64]. This data support the hypothesis that the threshold of [Ca^2+^]_int_ necessary for T cell proliferation seems to be low and proliferation does not require high [Ca^2+^]_int_ levels [65]. However, we did observe declined proliferative capacity in the first three days during stimulation which has been lifted from day four. Similar observations of declined proliferation have already been reported for CD4^+^ and CD8^+^ T cells from elderly mice and humans [4, 26, 61, 65–67]. Maybe, this decelerated proliferation benefits the cytotoxic capacity of CD8^+^ T cells and represents an elaborate adaptation during aging process.

Apoptotic deletion of activated T cells is an essential physiological process to terminate the immune response and the control of the overall number of immunocompetent cells [68, 69]. Ca^2+^ signaling plays a critical role for the initiation and effectuation of cell death [70–73]. Studies about apoptosis in aging are controversial and have shown increased apoptosis in T cells while others have observed a decrease or no effect [74–78]. Beside STIM- and Orai-mediated Ca^2+^ signals being crucial for T cell cytotoxicity and proliferation, excessive or prolonged Ca^2+^ entry can also lead to cell death [62]. Since it has been shown that Orai1 deficiency renders T cells resistant to death upon long-term exposure to anti-CD3 and anti-CD28 [62], the decreased Ca^2+^ influx in CD8^+^ T cells from elderly individuals could provide potential protection against increased apoptosis.

Although the signaling machinery in T cells is extremely complicated and many steps remain to be clarified, age-related changes in Ca^2+^ entry may be one important cause of cell-mediated immune response decline with aging. In this study we were able to demonstrate the contribution of Orai, STIM and PMCA in this multifaceted network of channels and pumps in Ca^2+^ homeostasis at molecular levels. Additional studies are required to clarify the influence of the reduced Ca^2+^ signaling in the context of CD8^+^ T cells cytotoxicity as one of the main killers in our body.

## MATERIALS AND METHODS

### Abs and reagents

If not mentioned otherwise, chemicals were purchased from Sigma Aldrich and antibodies from Biolegend. Antibodies used in our experiments include PerCP-labeled anti-mouse CD3 (145-2C11), Pacific Blue-labeled anti-mouse CD4 (RM4-5), FITC-labeled anti-mouse CD8a (53-6.7), PE-labeled anti-mouse/human CD44 (IM7), APC-labeled anti-mouse CD62L (MEL-14) and LEAF™ Purified anti-mouse CD3ε (1452C11).

### Mice

C57BL6/J mice were bread in our own colony from stock purchased from Charles River Laboratories. Only female mice were used between 12 and 24 weeks (adult mice) or between 78 and 102 weeks (elderly mice). Mice with splenomegaly (spleen to body weight ratio above 0.6) or macroscopically visible tumors were excluded from the studies. Mice were housed under specific pathogen-free conditions. At the designated times, animals were sacrificed by cervical dislocation and their spleens were harvested. Splenocytes were isolated by pushing the spleen through a 70 μm cell strainer (Corning®) and depleting the erythrocytes by a hypoosmolar solution. All animals used for this study were sacrificed and their organs harvested in compliance with the German Animal Protection Law (Tierschutzgesetz, §11, Abs.1 Nr.1).

### T cell culture and stimulation

CD8^+^ T cells were negatively isolated from murine splenocytes by using the Dynabeads™ Untouched™ Mouse CD8 Cells Kit (ThermoFisher). Murine CD8^+^ T cells were cultured in AIM V medium, supplemented with 10 % FCS, 100 U/ml IL-2, and 50 μM β-Mercaptoethanol, without stimulus. For the stimulation, the above mentioned medium was supplemented with Dynabeads™ Mouse T-Activator CD3/CD28 for T-Cell Expansion and Activation (ThermoFisher). The stimulated CD8^+^ T cells were kept in the medium with a 5:4 cell-to-bead ratio at 37 °C and 5 % CO_2_ for up to 4 days.

### Flow cytometry and cell sorting

Splenocytes and CD8^+^ T cells of adult and elderly mice were stained with the antibodies listed in ‘Abs and reagents’ and incubated for 20 min in the dark at room temperature. Stained samples were acquired using a FACSVerse™ (BD Biosciences) flow cytometer, and acquired data were analyzed with FlowJo software (FlowJo, LLC). For the sorted subtypes, stained 72 h stimulated CD8^+^ T cells were sorted on a FACSAria™III (BD Biosciences) sorter and left to rest from the sorting procedure for at least 4 h.

### Quantitative real-time PCR

Total RNA from CD8^+^ T cells was extracted by phenol-chloroform extraction. The concentration of intact total RNA was measured with a BioPhotometer (Eppendorf). Real-time PCR was performed with a Real Time System CFX 96/Thermal Cycler C1000 (BioRad). Relative expression levels were calculated using the ΔCq method (2^-ΔCq^). Expression of STIMs and Orais was normalized to the average mRNA levels of the housekeeping genes HPRT1 and TBP. Primers for QuantiTect Primer Assays were purchased from QIAGEN.

### QuantiTect primers

**Table:** 

**Target gene**	**Product**	**Cat. No.**
Orai1	Mm_Orai1_1_SG	QT00285775
Orai2	Mm_Orai2_1_SG	QT00304738
Orai3	Mm_Orai3_1_SG	QT00255598
STIM1	Mm_Stim1_1_SG	QT00105119
STIM2	Mm_Stim2_1_SG	QT00289009
PMCA1	Mm_Atp2b1_1_SG	QT01072106
PMCA4	Mm_Atp2b4_2_SG	QT01076271
HPRT1	Mm_Hprt_1_SG	QT00166768
TBP	Mm_Tbp_1_SG	QT00198443

### Western blot analysis

CD8^+^ T cells were collected directly after isolation (untouched) or after 72 h of stimulation (stimulated). Equivalent amounts of proteins were separated by 12–14 % SDS-PAGE and transferred to nitrocellulose membrane using a transblot electrophoresis transfer cell (Fisherbrand). Primary antibodies against Orai1, STIM1 and 2 were purchased from Proteintech. Primary antibody for GAPDH as reference was purchased from Cell Signaling. Secondary anti-rabbit antibody was purchased from GE Healthcare. ECL reagent (Amersham) was used for immunoblot detection. Densitometric quantification of Western blot data was done with Quantity one software (Bio-Rad).

### Fluorescence-based Ca^2+^-imaging

Ca^2+^-Imaging was performed according to Alansary, Kilch et al. paper [79]. Briefly, murine CD8^+^ T cells were loaded in AIM V medium with 1 μM Fura2-AM for 30 min at room temperature and allowed to attach to polyornithine-coated glass coverslips for 15 min. All experiments were carried out at room temperature in self-built perfusion chambers with low volume and high solution exchange rate. The external Ca^2+^ Ringer solution contained (in mM): 155 NaCl, 2 MgCl_2_, 10 glucose, 5 HEPES and 0.5 or 2 CaCl_2_ (0.5 / 2 Ca^2+^ Ringer) or no CaCl_2_, but 1 EGTA and 3 MgCl_2_ instead (0 Ca^2+^ Ringer). The pH was adjusted to 7.4 with NaOH. Images were analyzed with VisiView software (Visitron). Quantification of the trace shows the ratio (340 nm/380 nm) corresponding to Ca^2+^ influx peak and plateau and Ca^2+^ influx and efflux rate as ratio (340 nm/380 nm)s^-1^. Parameters analyzed were the influx peak, as maximal Ca^2+^ signal reached after TG application after (re-)adding of Ca^2+^, and the average plateau. The plateau mirrors the balance between influx and efflux and was analyzed before the application of 0 mM Ca^2+^ (Ca^2+^ removal).

### Patch-clamp measurements

Patch-clamp experiments were performed in the tight-seal whole-cell configuration at RT. Voltage ramps of 50 ms duration spanning a range of –100 to +100 mV were delivered from a holding potential of 0 mV at a rate of 0.5 Hz. All voltages were corrected for a liquid junction potential of 10 mV. Currents were filtered at 2.9 kHz and digitized at 100 μs intervals. Capacitive currents were determined and corrected before each ramp. Statistical errors of averaged data are given as means ± SEM with n determinations. Standard external solutions were as follows (in mM): 120 NaCl, 2.8 KCl, 2 MgCl_2_, 10 CaCl_2_, 10 CsCl, 10 HEPES, 10 glucose, pH 7.2 with NaOH, 305 mOsm. Standard internal solutions were as follows (in mM): 120 Cs-glutamate, 10 Cs-BAPTA, 3 MgCl_2_, 0 CaCl_2_, 10 HEPES, pH 7.2 with CsOH, 298 mOsm.

### Real-time killing assay

The real-time killing assays were carried out as described in Kummerow et al. 2014 [80]. Briefly, P815 mastocytoma cells were loaded with 500 nM calcein-AM in AIM V medium containing 10 mM HEPES at room temperature for 15 minutes. The loaded P815 cells were then settled at 2.5×10^4^ cells per well into black 96-well plates with clear-bottoms (353219, Corning, Amsterdam, Netherlands). CD8^+^ T cells were pulsed with 2 μg/ml anti-CD3ε antibody and subsequently added onto the P815 cells at a 20:1 effector to target ratio. Target lysis was measured either in an M200 Infinite plate reader (Tecan, Crailsheim, Germany) or a Genios Pro (Tecan) using bottom reading function at 37°C. The quantification of free Ca^2+^ concentration in AIM V medium supplemented with different amounts of Ca^2+^ or EGTA was done as previously described in Zhou et al. 2018 [12].

### Statistical analysis

All values are given as mean ± SEM or SD. Data were analyzed using VisiView (Visitron), Microsoft Excel (Microsoft), Igor Pro (Wavemetrics), Image Lab™ (Bio-Rad) and GraphPad (GraphPad Software Inc.). Rate constants (*k* values) of iso-cells were calculated for each trace by exponential decay analysis after removal of external Ca^2+^. Significances of data were calculated with an unpaired two-sided Student’s t-test if Gaussian distribution was given. If no Gaussian distribution was given, data were analyzed with the nonparametric Mann-Whitney test. For multi-parameter analysis data were analyzed with ANOVA. Degrees of significance were set at * p < 0.05, ** p < 0.01, *** p < 0.001 and **** p < 0.0001.

## Supplementary Material

Supplementary Figures

Supplementary Tables

## References

[1] Weyand CM, Goronzy JJ. Aging of the Immune System. Mechanisms and Therapeutic Targets. Ann Am Thorac Soc. 2016 (Suppl 5); 13:S422–28. 10.1513/AnnalsATS.201602-095AW 28005419 PMC5291468

[2] Gavazzi G, Krause KH. Ageing and infection. Lancet Infect Dis. 2002; 2:659–66. 10.1016/S1473-3099(02)00437-1 12409046

[3] Nikolich-Zugich J. Ageing and life-long maintenance of T-cell subsets in the face of latent persistent infections. Nat Rev Immunol. 2008; 8:512–22. 10.1038/nri2318 18469829 PMC5573867

[4] Nikolich-Žugich J, Li G, Uhrlaub JL, Renkema KR, Smithey MJ. Age-related changes in CD8 T cell homeostasis and immunity to infection. Semin Immunol. 2012; 24:356–64. 10.1016/j.smim.2012.04.009 22554418 PMC3480557

[5] Feske S, Wulff H, Skolnik EY. Ion channels in innate and adaptive immunity. Annu Rev Immunol. 2015; 33:291–353. 10.1146/annurev-immunol-032414-112212 25861976 PMC4822408

[6] Trebak M, Kinet JP. Calcium signalling in T cells. Nat Rev Immunol. 2019; 19:154–69. 10.1038/s41577-018-0110-7 30622345 PMC6788797

[7] Berridge MJ, Lipp P, Bootman MD. The versatility and universality of calcium signalling. Nat Rev Mol Cell Biol. 2000; 1:11–21. 10.1038/35036035 11413485

[8] Dolmetsch RE, Lewis RS, Goodnow CC, Healy JI. Differential activation of transcription factors induced by Ca2+ response amplitude and duration. Nature. 1997; 386:855–58. 10.1038/386855a0 9126747

[9] Zheng Y, Zha Y, Gajewski TF. Molecular regulation of T-cell anergy. EMBO Rep. 2008; 9:50–55. 10.1038/sj.embor.7401138 18174897 PMC2246614

[10] Srinivasan M, Frauwirth KA. Peripheral tolerance in CD8+ T cells. Cytokine. 2009; 46:147–59. 10.1016/j.cyto.2009.01.010 19268604

[11] Kuklina EM. Molecular mechanisms of T-cell anergy. Biochemistry (Mosc). 2013; 78:144–56. 10.1134/S000629791302003X 23581985

[12] Zhou X, Friedmann KS, Lyrmann H, Zhou Y, Schoppmeyer R, Knörck A, Mang S, Hoxha C, Angenendt A, Backes CS, Mangerich C, Zhao R, Cappello S, et al. A calcium optimum for cytotoxic T lymphocyte and natural killer cell cytotoxicity. J Physiol. 2018; 596:2681–98. 10.1113/JP274964 29368348 PMC6046087

[13] Schwarz EC, Qu B, Hoth M. Calcium, cancer and killing: the role of calcium in killing cancer cells by cytotoxic T lymphocytes and natural killer cells. Biochim Biophys Acta. 2013; 1833:1603–11. 10.1016/j.bbamcr.2012.11.016 23220009

[14] Feske S, Skolnik EY, Prakriya M. Ion channels and transporters in lymphocyte function and immunity. Nat Rev Immunol. 2012; 12:532–47. 10.1038/nri3233 22699833 PMC3670817

[15] Lis A, Peinelt C, Beck A, Parvez S, Monteilh-Zoller M, Fleig A, Penner R. CRACM1, CRACM2, and CRACM3 are store-operated Ca2+ channels with distinct functional properties. Curr Biol. 2007; 17:794–800. 10.1016/j.cub.2007.03.065 17442569 PMC5663639

[16] DeHaven WI, Smyth JT, Boyles RR, Putney JW Jr. Calcium inhibition and calcium potentiation of Orai1, Orai2, and Orai3 calcium release-activated calcium channels. J Biol Chem. 2007; 282:17548–56. 10.1074/jbc.M611374200 17452328

[17] Parekh AB. Decoding cytosolic Ca2+ oscillations. Trends Biochem Sci. 2011; 36:78–87. 10.1016/j.tibs.2010.07.013 20810284

[18] Gross SA, Wissenbach U, Philipp SE, Freichel M, Cavalié A, Flockerzi V. Murine ORAI2 splice variants form functional Ca2+ release-activated Ca2+ (CRAC) channels. J Biol Chem. 2007; 282:19375–84. 10.1074/jbc.M701962200 17463004

[19] Darbellay B, Arnaudeau S, Bader CR, Konig S, Bernheim L. STIM1L is a new actin-binding splice variant involved in fast repetitive Ca2+ release. J Cell Biol. 2011; 194:335–46. 10.1083/jcb.201012157 21788372 PMC3144404

[20] Miederer AM, Alansary D, Schwär G, Lee PH, Jung M, Helms V, Niemeyer BA. A STIM2 splice variant negatively regulates store-operated calcium entry. Nat Commun. 2015; 6:6899. 10.1038/ncomms7899 25896806 PMC4411291

[21] Wissenbach U, Philipp SE, Gross SA, Cavalié A, Flockerzi V. Primary structure, chromosomal localization and expression in immune cells of the murine ORAI and STIM genes. Cell Calcium. 2007; 42:439–46. 10.1016/j.ceca.2007.05.014 17659338

[22] Cahalan MD, Chandy KG. The functional network of ion channels in T lymphocytes. Immunol Rev. 2009; 231:59–87. 10.1111/j.1600-065X.2009.00816.x 19754890 PMC3133616

[23] Launay P, Cheng H, Srivatsan S, Penner R, Fleig A, Kinet JP. TRPM4 regulates calcium oscillations after T cell activation. Science. 2004; 306:1374–77. 10.1126/science.1098845 15550671

[24] Brini M, Carafoli E. The plasma membrane Ca²+ ATPase and the plasma membrane sodium calcium exchanger cooperate in the regulation of cell calcium. Cold Spring Harb Perspect Biol. 2011; 3:3. 10.1101/cshperspect.a004168 21421919 PMC3039526

[25] Miller RA, Jacobson B, Weil G, Simons ER. Diminished calcium influx in lectin-stimulated T cells from old mice. J Cell Physiol. 1987; 132:337–42. 10.1002/jcp.1041320220 3497930

[26] Grossmann A, Maggio-Price L, Jinneman JC, Rabinovitch PS. Influence of aging on intracellular free calcium and proliferation of mouse T-cell subsets from various lymphoid organs. Cell Immunol. 1991; 135:118–31. 10.1016/0008-8749(91)90259-E 1826862

[27] Pettan-Brewer C, Treuting PM. Practical pathology of aging mice. Pathobiol Aging Age Relat Dis. 2011; 1:1. 10.3402/pba.v1i0.7202 22953032 PMC3417704

[28] Pinchuk LM, Filipov NM. Differential effects of age on circulating and splenic leukocyte populations in C57BL/6 and BALB/c male mice. Immun Ageing. 2008; 5:1. 10.1186/1742-4933-5-1 18267021 PMC2268915

[29] Quinn KM, Fox A, Harland KL, Russ BE, Li J, Nguyen TH, Loh L, Olshanksy M, Naeem H, Tsyganov K, Wiede F, Webster R, Blyth C, et al. Age-Related Decline in Primary CD8^+^ T Cell Responses Is Associated with the Development of Senescence in Virtual Memory CD8^+^ T Cells. Cell Rep. 2018; 23:3512–24. 10.1016/j.celrep.2018.05.057 29924995

[30] Sarkadi B, Tordai A, Homolya L, Scharff O, Gárdos G. Calcium influx and intracellular calcium release in anti-CD3 antibody-stimulated and thapsigargin-treated human T lymphoblasts. J Membr Biol. 1991; 123:9–21. 10.1007/BF01993958 1723105

[31] Shiku H, Kisielow P, Bean MA, Takahashi T, Boyse EA, Oettgen HF, Old LJ. Expression of T-cell differentiation antigens on effector cells in cell-mediated cytotoxicity in vitro. Evidence for functional heterogeneity related to the surface phenotype of T cells. J Exp Med. 1975; 141:227–41. 10.1084/jem.141.1.227 1078839 PMC2190510

[32] Hogan PG, Lewis RS, Rao A. Molecular basis of calcium signaling in lymphocytes: STIM and ORAI. Annu Rev Immunol. 2010; 28:491–533. 10.1146/annurev.immunol.021908.132550 20307213 PMC2861828

[33] Vaeth M, Yang J, Yamashita M, Zee I, Eckstein M, Knosp C, Kaufmann U, Karoly Jani P, Lacruz RS, Flockerzi V, Kacskovics I, Prakriya M, Feske S. ORAI2 modulates store-operated calcium entry and T cell-mediated immunity. Nat Commun. 2017; 8:14714. 10.1038/ncomms14714 28294127 PMC5355949

[34] Hoover PJ, Lewis RS. Stoichiometric requirements for trapping and gating of Ca2+ release-activated Ca2+ (CRAC) channels by stromal interaction molecule 1 (STIM1). Proc Natl Acad Sci USA. 2011; 108:13299–304. 10.1073/pnas.1101664108 21788510 PMC3156176

[35] Li Z, Liu L, Deng Y, Ji W, Du W, Xu P, Chen L, Xu T. Graded activation of CRAC channel by binding of different numbers of STIM1 to Orai1 subunits. Cell Res. 2011; 21:305–15. 10.1038/cr.2010.131 20838418 PMC3193435

[36] Donnadieu E, Bismuth G, Trautmann A. Calcium fluxes in T lymphocytes. J Biol Chem. 1992; 267:25864–72. 1464601

[37] Bautista DM, Hoth M, Lewis RS. Enhancement of calcium signalling dynamics and stability by delayed modulation of the plasma-membrane calcium-ATPase in human T cells. J Physiol. 2002; 541:877–94. 10.1113/jphysiol.2001.016154 12068047 PMC2290354

[38] Bautista DM, Lewis RS. Modulation of plasma membrane calcium-ATPase activity by local calcium microdomains near CRAC channels in human T cells. J Physiol. 2004; 556:805–17. 10.1113/jphysiol.2003.060004 14966303 PMC1665005

[39] Mattson MP. Calcium and neurodegeneration. Aging Cell. 2007; 6:337–50. 10.1111/j.1474-9726.2007.00275.x 17328689

[40] Bezprozvanny IB. Calcium signaling and neurodegeneration. Acta Naturae. 2010; 2:72–82. 10.32607/20758251-2010-2-1-72-80 22649630 PMC3347543

[41] Pchitskaya E, Popugaeva E, Bezprozvanny I. Calcium signaling and molecular mechanisms underlying neurodegenerative diseases. Cell Calcium. 2018; 70:87–94. 10.1016/j.ceca.2017.06.008 28728834 PMC5748019

[42] Popugaeva E, Pchitskaya E, Bezprozvanny I. Dysregulation of Intracellular Calcium Signaling in Alzheimer's Disease. Antioxid Redox Signal. 2018; 29:1176–88. 10.1089/ars.2018.7506 29890840 PMC6157344

[43] Bose T, Cieślar-Pobuda A, Wiechec E. Role of ion channels in regulating Ca² ^+^ homeostasis during the interplay between immune and cancer cells. Cell Death Dis. 2015; 6:e1648. 10.1038/cddis.2015.23 25695601 PMC4669790

[44] Dorshkind K, Montecino-Rodriguez E, Signer RA. The ageing immune system: is it ever too old to become young again? Nat Rev Immunol. 2009; 9:57–62. 10.1038/nri2471 19104499

[45] Kline KA, Bowdish DM. Infection in an aging population. Curr Opin Microbiol. 2016; 29:63–67. 10.1016/j.mib.2015.11.003 26673958

[46] Giefing-Kröll C, Berger P, Lepperdinger G, Grubeck-Loebenstein B. How sex and age affect immune responses, susceptibility to infections, and response to vaccination. Aging Cell. 2015; 14:309–21. 10.1111/acel.12326 25720438 PMC4406660

[47] de Saint Basile G, Ménasché G, Fischer A. Molecular mechanisms of biogenesis and exocytosis of cytotoxic granules. Nat Rev Immunol. 2010; 10:568–79. 10.1038/nri2803 20634814

[48] Lieberman J. The ABCs of granule-mediated cytotoxicity: new weapons in the arsenal. Nat Rev Immunol. 2003; 3:361–70. 10.1038/nri1083 12766758

[49] Barry M, Bleackley RC. Cytotoxic T lymphocytes: all roads lead to death. Nat Rev Immunol. 2002; 2:401–09. 10.1038/nri819 12093006

[50] Fierro L, Parekh AB. Substantial depletion of the intracellular Ca2+ stores is required for macroscopic activation of the Ca2+ release-activated Ca2+ current in rat basophilic leukaemia cells. J Physiol. 2000; 522:247–57. 10.1111/j.1469-7793.2000.t01-1-00247.x 10639101 PMC2269755

[51] Hoth M, Penner R. Calcium release-activated calcium current in rat mast cells. J Physiol. 1993; 465:359–86. 10.1113/jphysiol.1993.sp019681 8229840 PMC1175434

[52] Vogel C, Marcotte EM. Insights into the regulation of protein abundance from proteomic and transcriptomic analyses. Nat Rev Genet. 2012; 13:227–32. 10.1038/nrg3185 22411467 PMC3654667

[53] Niemeyer BA. Changing calcium: CRAC channel (STIM and Orai) expression, splicing, and posttranslational modifiers. Am J Physiol Cell Physiol. 2016; 310:C701–09. 10.1152/ajpcell.00034.2016 26911279

[54] Williams RT, Manji SS, Parker NJ, Hancock MS, Van Stekelenburg L, Eid JP, Senior PV, Kazenwadel JS, Shandala T, Saint R, Smith PJ, Dziadek MA. Identification and characterization of the STIM (stromal interaction molecule) gene family: coding for a novel class of transmembrane proteins. Biochem J. 2001; 357:673–85. 10.1042/bj3570673 11463338 PMC1221997

[55] Scrimgeour N, Litjens T, Ma L, Barritt GJ, Rychkov GY. Properties of Orai1 mediated store-operated current depend on the expression levels of STIM1 and Orai1 proteins. J Physiol. 2009; 587:2903–18. 10.1113/jphysiol.2009.170662 19403622 PMC2718249

[56] Frisch J, Angenendt A, Hoth M, Prates Roma L, Lis A. STIM-Orai Channels and Reactive Oxygen Species in the Tumor Microenvironment. Cancers (Basel). 2019; 11:11. 10.3390/cancers11040457 30935064 PMC6520831

[57] Berridge MJ. Calcium signalling and cell proliferation. BioEssays. 1995; 17:491–500. 10.1002/bies.950170605 7575490

[58] Lu KP, Means AR. Regulation of the cell cycle by calcium and calmodulin. Endocr Rev. 1993; 14:40–58. 10.1210/edrv-14-1-40 8491154

[59] Shah GV, Rayford W, Noble MJ, Austenfeld M, Weigel J, Vamos S, Mebust WK. Calcitonin stimulates growth of human prostate cancer cells through receptor-mediated increase in cyclic adenosine 3′,5′-monophosphates and cytoplasmic Ca2+ transients. Endocrinology. 1994; 134:596–602. 10.1210/endo.134.2.8299557 8299557

[60] Hoth M. CRAC channels, calcium, and cancer in light of the driver and passenger concept. Biochim Biophys Acta. 2016; 1863:1408–17. 10.1016/j.bbamcr.2015.12.009 26705695

[61] Vaeth M, Maus M, Klein-Hessling S, Freinkman E, Yang J, Eckstein M, Cameron S, Turvey SE, Serfling E, Berberich-Siebelt F, Possemato R, Feske S. Store-Operated Ca(2+) Entry Controls Clonal Expansion of T Cells through Metabolic Reprogramming. Immunity. 2017; 47:664–79.e6. 10.1016/j.immuni.2017.09.003 29030115 PMC5683398

[62] Kim KD, Srikanth S, Yee MK, Mock DC, Lawson GW, Gwack Y. ORAI1 deficiency impairs activated T cell death and enhances T cell survival. J Immunol. 2011; 187:3620–30. 10.4049/jimmunol.1100847 21873530 PMC3178683

[63] McCarl CA, Khalil S, Ma J, Oh-hora M, Yamashita M, Roether J, Kawasaki T, Jairaman A, Sasaki Y, Prakriya M, Feske S. Store-operated Ca2+ entry through ORAI1 is critical for T cell-mediated autoimmunity and allograft rejection. J Immunol. 2010; 185:5845–58. 10.4049/jimmunol.1001796 20956344 PMC2974040

[64] Ma J, McCarl CA, Khalil S, Lüthy K, Feske S. T-cell-specific deletion of STIM1 and STIM2 protects mice from EAE by impairing the effector functions of Th1 and Th17 cells. Eur J Immunol. 2010; 40:3028–42. 10.1002/eji.201040614 21061435 PMC3517124

[65] Schwarz EC, Kummerow C, Wenning AS, Wagner K, Sappok A, Waggershauser K, Griesemer D, Strauss B, Wolfs MJ, Quintana A, Hoth M. Calcium dependence of T cell proliferation following focal stimulation. Eur J Immunol. 2007; 37:2723–33. 10.1002/eji.200737039 17899547

[66] Nikolich-Žugich J. Aging of the T cell compartment in mice and humans: from no naive expectations to foggy memories. J Immunol. 2014; 193:2622–29. 10.4049/jimmunol.1401174 25193936 PMC4157314

[67] Decman V, Laidlaw BJ, Dimenna LJ, Abdulla S, Mozdzanowska K, Erikson J, Ertl HC, Wherry EJ. Cell-intrinsic defects in the proliferative response of antiviral memory CD8 T cells in aged mice upon secondary infection. J Immunol. 2010; 184:5151–59. 10.4049/jimmunol.0902063 20368274

[68] Oehm A, Behrmann I, Falk W, Pawlita M, Maier G, Klas C, Li-Weber M, Richards S, Dhein J, Trauth BC, et al. Purification and molecular cloning of the APO-1 cell surface antigen, a member of the tumor necrosis factor/nerve growth factor receptor superfamily. Sequence identity with the Fas antigen. J Biol Chem. 1992; 267:10709–15. 1375228

[69] Zhan Y, Carrington EM, Zhang Y, Heinzel S, Lew AM. Life and Death of Activated T Cells: How Are They Different from Naïve T Cells? Front Immunol. 2017; 8:1809. 10.3389/fimmu.2017.01809 29326701 PMC5733345

[70] Verkhratsky A. Calcium and cell death. Subcell Biochem. 2007; 45:465–80. 10.1007/978-1-4020-6191-2_17 18193648

[71] Orrenius S, Zhivotovsky B, Nicotera P. Regulation of cell death: the calcium-apoptosis link. Nat Rev Mol Cell Biol. 2003; 4:552–65. 10.1038/nrm1150 12838338

[72] Berridge MJ, Bootman MD, Lipp P. Calcium—a life and death signal. Nature. 1998; 395:645–48. 10.1038/27094 9790183

[73] Qu B, Al-Ansary D, Kummerow C, Hoth M, Schwarz EC. ORAI-mediated calcium influx in T cell proliferation, apoptosis and tolerance. Cell Calcium. 2011; 50:261–69. 10.1016/j.ceca.2011.05.015 21665274

[74] Spaulding CC, Walford RL, Effros RB. The accumulation of non-replicative, non-functional, senescent T cells with age is avoided in calorically restricted mice by an enhancement of T cell apoptosis. Mech Ageing Dev. 1997; 93:25–33. 10.1016/S0047-6374(96)01808-8 9089568

[75] Chrest FJ, Buchholz MA, Kim YH, Kwon TK, Nordin AA. Anti-CD3-induced apoptosis in T-cells from young and old mice. Cytometry. 1995; 20:33–42. 10.1002/cyto.990200107 7600898

[76] Zhou T, Edwards CK 3rd, Mountz JD. Prevention of age-related T cell apoptosis defect in CD2-fas-transgenic mice. J Exp Med. 1995; 182:129–37. 10.1084/jem.182.1.129 7540646 PMC2192099

[77] Gupta S. Molecular mechanisms of apoptosis in the cells of the immune system in human aging. Immunol Rev. 2005; 205:114–29. 10.1111/j.0105-2896.2005.00261.x 15882349

[78] Sikora E. Activation-induced and damage-induced cell death in aging human T cells. Mech Ageing Dev. 2015; 151:85–92. 10.1016/j.mad.2015.03.011 25843236

[79] Alansary D, Kilch T, Holzmann C, Peinelt C, Hoth M, Lis A. The minimal requirements to use calcium imaging to analyze ICRAC. Cold Spring Harb Protoc. 2014; 2014:638–42. 10.1101/pdb.prot073262 24890204

[80] Kummerow C, Schwarz EC, Bufe B, Zufall F, Hoth M, Qu B. A simple, economic, time-resolved killing assay. Eur J Immunol. 2014; 44:1870–72. 10.1002/eji.201444518 24599783

