# Supplementary material for: Orai, STIM, and PMCA contribute to reduced calcium signal generation in CD8+ T cells of elderly mice
Source: Aging (Albany NY). 2020 Feb 12;12(4):3266–86. doi: 10.18632/aging.102809 (PMC7066920; doi:10.18632/aging.102809)
Supplement: Supplementary Figures [file aging-12-102809-s002..pdf]

SUPPLEMENTARY FIGURES

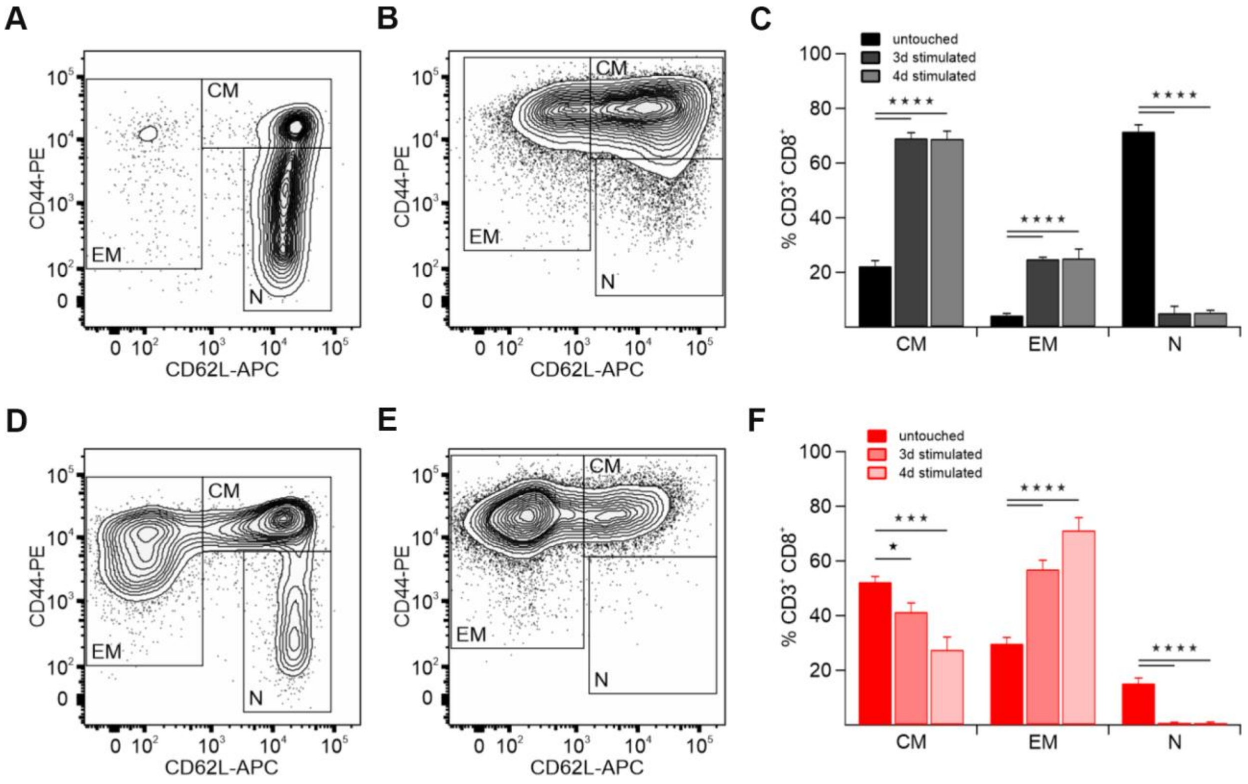

Supplementary Figure 1. CD8⁺ T cell subtype distribution shifts from more naïve to more memory cells in elderly mice. Exemplary contour plots of untouched CD8⁺ T cells from adult (**A**) and elderly (**B**) and stimulated CD8⁺ T cells from adult (**D**) and elderly mice (**E**). (**C**) Subtype distribution from untouched (n = 35), three (n = 20) and four days stimulated (n = 10) CD8⁺ T cells from adult mice. (**F**) Subtype distribution from untouched (n = 31), three (n = 20) and four days stimulated (n = 8) CD8⁺ T cells from elderly mice. Data obtained are presented as mean ± SEM. * $p < 0.05$, ** $p < 0.01$, *** $p < 0.001$, **** $p < 0.0001$.

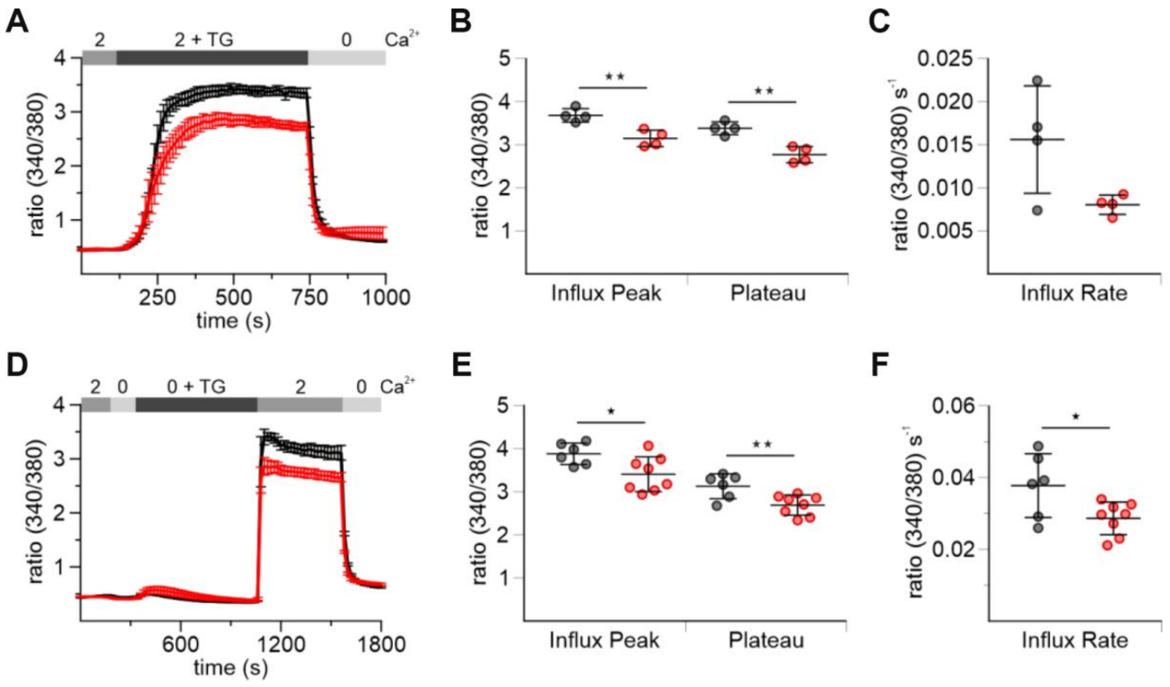

Supplementary Figure 2. Untouched CD8$^+$ T cells from elderly mice exhibit reduced thapsigargin (TG)-induced Ca^{2+} signals. (**A**) Fura2-AM based Ca^{2+} Imaging with 1 µM TG as stimulus applied in the presence of 2 mM $[Ca^{2+}]_{ext}$ (combined Ca^{2+} protocol) of CD8$^+$ T cells from adult (black, n = 4) and elderly (red, n = 4) mice. Scatter dot plot in (**B**) displays the corresponding statistic of Ca^{2+} influx peak and Ca^{2+} plateau and in (**C**) the corresponding influx rates. (**D**) Ca^{2+} Imaging with 1 µM TG applied in the absence of $[Ca^{2+}]_{ext}$ before re-addition of 2 mM Ca^{2+} (re-addition protocol) of CD8$^+$ T cells from adult (black, n = 6) and elderly (red, n = 8) mice. The scatter dot plot in (**E**) displays the corresponding statistic of Ca^{2+} influx peak and Ca^{2+} plateau and (**F**) the corresponding influx rates. Ca^{2+} data are presented as mean ± SEM. Scatter dot plots are presented as mean ± SD. * $p < 0.05$, ** $p < 0.01$, *** $p < 0.001$, **** $p < 0.0001$.

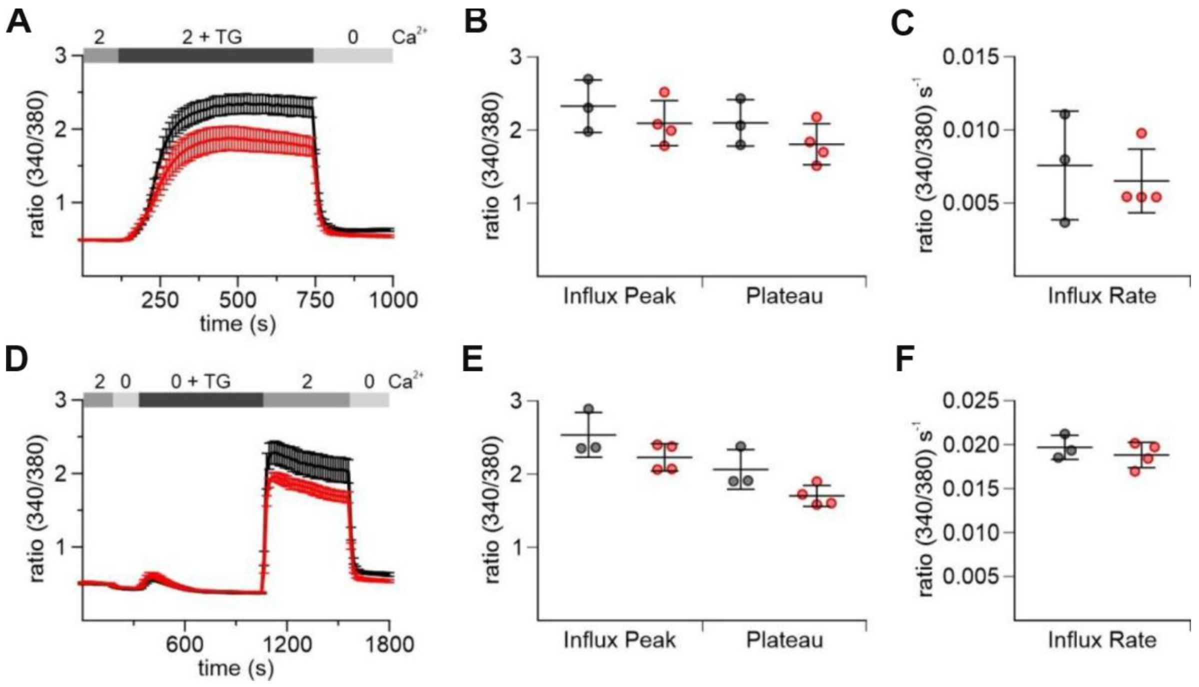

Supplementary Figure 3. Stimulated CD8+ T cells from elderly mice exhibit reduced thapsigargin (TG)-induced Ca2+ signals. (**A**) Fura2-AM based Ca2+ Imaging with 1 µM TG as stimulus applied in the presence of 2 mM [Ca2+]ext (combined Ca2+ protocol) of CD8+ T cells from adult (black, n = 3) and elderly (red, n = 4) mice. The scatter dot plot in (**B**) displays the corresponding statistic of Ca2+ influx peak and Ca2+ plateau and in (**C**) the corresponding influx rates. (**D**) Ca2+ Imaging with 1 µM TG applied in the absence of [Ca2+]ext before re-addition of 2 mM Ca2+ (re-addition protocol) of CD8+ T cells from adult (black, n = 3) and elderly (red, n = 4) mice. The scatter dot plot in (**E**) displays the corresponding statistic of Ca2+ influx peak and Ca2+ plateau and (**F**) the corresponding influx rates. Ca2+ signalling curves are presented as mean ± SEM. Scatter dot plots are presented as mean ± SD. * $p < 0.05$, ** $p < 0.01$, *** $p < 0.001$, **** $p < 0.0001$.

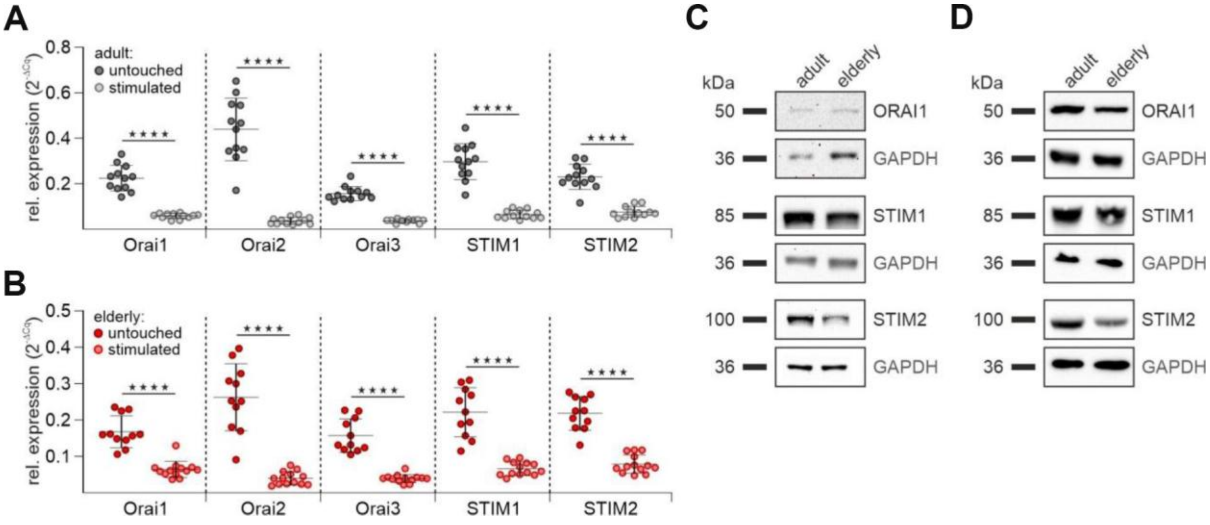

Supplementary Figure 4. mRNA expression of SOCE components declines significantly with stimulation/activation of CD8⁺ T cells from both age groups. (**A**) Relative mRNA expressions of Orai1, 2 and 3 and STIM1 and 2 of untouched (dark grey, n = 12) and stimulated (light grey, n = 12) CD8⁺ T cells from adult mice. (**B**) Relative mRNA expressions of Orai1, 2 and 3 and STIM1 and 2 of untouched (dark red, n = 11) and stimulated (light red, n = 13) CD8⁺ T cells from elderly mice. Representative Western blots of SOCE components from untouched (**C**) and stimulated (**D**) CD8⁺ T cells. Scatter dot plots are presented as mean ± SD. * $p < 0.05$, ** $p < 0.01$, *** $p < 0.001$, **** $p < 0.0001$.
