# Supplementary material for: Orai, STIM, and PMCA contribute to reduced calcium signal generation in CD8+ T cells of elderly mice
Source: Aging (Albany NY). 2020 Feb 12;12(4):3266–86. doi: 10.18632/aging.102809 (PMC7066920; doi:10.18632/aging.102809)
Supplement: Supplementary Tables [file aging-12-102809-s001..pdf]

SUPPLEMENTARY TABLES

Supplementary Table 1. Quantification of Ca^{2+} signal parameters (ratio 340/380 ± SEM) for combined Ca^{2+} measurements with 0.5 mM Ca^{2+} in the external solution as shown in Figures 1A, 3A and Supplementary Figure 5A, 5G.

	Basal Ca^{2+}	Influx Peak	Ca^{2+} Plateau	Influx Rate
**adult untouched**	0.41572 (± 0.00724)	3.35034 (± 0.06840)	2.86868 (± 0.11545)	0.01493 (± 0.00325)
**elderly untouched**	0.43849 (± 0.00963)	2.74772 (± 0.16623)	2.29437 (± 0.17377)	0.00943 (± 0.00138)
**adult stimulated**	0.44257 (± 0.00828)	1.58579 (± 0.08134)	1.32114 (± 0.06486)	0.00618 (± 0.00135)
**elderly stimulated**	0.45081 (± 0.00674)	1.22973 (± 0.11855)	0.99628 (± 0.10602)	0.00411 (± 0.00030)
**norm. adult sorted CM/EM**	1	1	1	1
**norm. elderly sorted CM**	1.00521 (± 0.01682)	0.76461 (± 0.04269)	0.76402 (± 0.03756)	0.66231 (± 0.06913)
**norm. elderly sorted EM**	0.97606 (± 0.00743)	0.89495 (± 0.04126)	0.90768 (± 0.03429)	0.77298 (± 0.09990)

Supplementary Table 2. Quantification of Ca^{2+} signal parameters (ratio 340/380 ± SEM) for re-addition measurements with 0.5 mM Ca^{2+} in the external solution as shown in Figures 1B, 3B and Supplementary Figure 5B, 5H.

	Basal Ca^{2+}	TG Peak	Influx Peak	Ca^{2+} Plateau	Influx Rate
**adult untouched**	0.40805 (± 0.00360)	0.54841 (± 0.01312)	3.44848 (± 0.12020)	2.71912 (± 0.09117)	0.04126 (± 0.00299)
**elderly untouched**	0.43947 (± 0.01381)	0.69555 (± 0.08362)	2.6784 (± 0.08594)	2.08323 (± 0.06764)	0.02871 (± 0.00205)
**adult stimulated**	0.49956 (± 0.02458)	0.55749 (± 0.01406)	1.73206 (± 0.08618)	1.26592 (± 0.08696)	0.01808 (± 0.00189)
**elderly stimulated**	0.46913 (± 0.01081)	0.62148 (± 0.03382)	1.34722 (± 0.06802)	0.92057 (± 0.05860)	0.01210 (± 0.00064)
**norm. adult sorted CM/EM**	1	1	1	1	1
**norm. elderly sorted CM**	0.99123 (± 0.01456)	1.03256 (± 0.01979)	0.80305 (± 0.03007)	0.77923 (± 0.03169)	0.81061 (± 0.09589)
**norm. elderly sorted EM**	0.99405 (± 0.01283)	1.02691 (± 0.04218)	0.91613 (± 0.02254)	0.91261 (± 0.01533)	0.95864 (± 0.04460)

Supplementary Table 3. Quantification of Ca^{2+} signal parameters (ratio 340/380 ± SEM) of combined Ca^{2+} measurements with 2 mM Ca^{2+} in the external solution as shown in Supplementary Figures 2A, 3A.

	Basal Ca^{2+}	Influx Peak	Ca^{2+} Plateau	Influx Rate
**adult untouched**	0.44246 (± 0.01405)	3.67726 (± 0.07794)	3.37786 (± 0.07495)	0.01558 (± 0.00312)
**elderly untouched**	0.45475 (± 0.01841)	3.18998 (± 0.08738)	2.77220 (± 0.07289)	0.01403 (± 0.00602)
**adult stimulated**	0.48054 (± 0.00932)	2.32779 (± 0.20696)	2.09853 (± 0.18375)	0.00757 (± 0.00214)
**elderly stimulated**	0.49030 (± 0.01157)	2.09466 (± 0.15442)	1.80740 (± 0.14058)	0.00651 (± 0.00109)

Supplementary Table 4. Quantification of Ca^{2+} signal parameters (ratio 340/380 ± SEM) for re-addition measurements with 2 mM Ca^{2+} in the external solution as shown in Supplementary Figures 2D, 3D.

	Basal Ca^{2+}	TG Peak	Influx Peak	Ca^{2+} Plateau	Influx Rate
**adult untouched**	0.44743 (± 0.01368)	0.52506 (± 0.01526)	3.87886 (± 0.10149)	3.12665 (± 0.11758)	0.03775 (± 0.00362)
**elderly untouched**	0.44508 (± 0.00836)	0.62696 (± 0.07810)	3.40563 (± 0.14274)	2.68961 (± 0.08306)	0.02863 (± 0.00161)
**adult stimulated**	0.49982 (± 0.01600)	0.55386 (± 0.00431)	2.53691 (± 0.17716)	2.06464 (± 0.15648)	0.01970 (± 0.00080)
**elderly stimulated**	0.51258 (± 0.01150)	0.64717 (± 0.03941)	2.22903 (± 0.09299)	1.70259 (± 0.07201)	0.01882 (± 0.00072)

Supplementary Table 5. Quantification of rate constant (*k*), calculated as 1/tau (sec^{-1} ± SEM), extract from global Ca^{2+} measurements with 0.5 mM $[Ca^{2+}]_{ext}$ for untouched and stimulated $CD8^+$ T cells isolated from adult and elderly mice as shown in Figure 6B, 6E.

$[Ca^{2+}]_{int}$	untouched				stimulated			
	adult		elderly		adult		elderly	
	mean	cells	mean	cells	mean	cells	mean	cells
**0-0.5**	-	-	-	-	0. 02306 (± 0,00241)	18	0.03365 (± 0.00406)	59
**0.5-1**	0. 04083 (± 0.00551)	40	0.053413 (± 0.00361)	88	0. 03919 (± 0,00066)	963	0.04978 (± 0.00091)	947
**1-1.5**	0.048453 (± 0.00552)	35	0.060124 (± 0.00387)	74	0.048255 (± 0.00103)	468	0.06430 (± 0.00207)	295
**1.5-2**	0.05410 (± 0.0050)	60	0.05795 (± 0.00384)	128	0.05175 (± 0.00129)	262	0.06784 (± 0.00418)	118
**2-2.5**	0.05111 (± 0.00324)	383	0.06567 (± 0.00284)	247	0.06127 (± 0.00136)	333	0.0744 (± 0.00477)	56
**2.5-3**	0.05461 (± 0.00094)	627	0.07003 (± 0.00284)	101	-	-	-	-
**3-3.5**	0.05139 (± 0.00054)	145	0.05901 (± 0.00284)	26	-	-	-	-
